# Descending Necrotizing Mediastinitis by Streptococcus anginosus and Prevotella buccae in an Intensive Care Unit Patient: A Case Report

**DOI:** 10.7759/cureus.39703

**Published:** 2023-05-30

**Authors:** Monica R Pachar Flores, Rafael Andrade-Alegre, Erika Santiago, Lizbeth Sierra

**Affiliations:** 1 Infectious Diseases, Hospital Santo Tomas, Panama, PAN; 2 Infectious Diseases, Instituto Oncologico Nacional, Panama, PAN; 3 Thoracic Surgery Section, Hospital Santo Tomas, Panama, PAN; 4 Microbiology, Hospital Santo Tomas, Panama, PAN; 5 Intensive Care Unit, Hospital Santo Tomas, Panama, PAN

**Keywords:** deep neck space infection, video-assisted thoracoscopic surgery (vats), thoracic empyema, odontogenic infections, anaerobes, anaerobes of clinical importance, mediastinitis management, descending necrotizing mediastinitis

## Abstract

Necrotizing infections of deep neck spaces are a group of life-threatening infectious diseases acquired through trauma or as a descending infection from an odontogenic source. The isolation of pathogens is unusual because of the anaerobic nature of the infection; however, one way to achieve this is through the use of automated microbiological methods like matrix-assisted laser desorption/ionization and time-of-flight (MALDI-TOF) following standard microbiology protocols for analyzing samples from potential anaerobic infections.

We present a case of a patient without risk factors for descending necrotizing mediastinitis with isolation *of Streptococcus anginosus and Prevotella*​​​​​​​* buccae* managed at the intensive care unit with a multidisciplinary team. We present our approach and how we successfully treat this complicated infection.

## Introduction

Descending necrotizing mediastinitis (DNM) was described for the first time by Pearse in 1938 as an infectious disease originating from a dental or oropharyngeal focus with dissemination through the fascial planes to the mediastinum. Unusual sources described in the literature include tonsils, parotid glands, mastoiditis, and epiglottitis [1].

Thirteen separate compartments form the deep spaces of the neck. It is recognized that an infection that starts in one area of the neck can travel quickly within their respective fascia planes and spread. Due to limited space, edema happens quickly, and the progression of the infection is fast. This phenomenon has implications for the management and prognosis due to necrosis of surrounding tissue, airway compromise, and dissemination to continuous compartments, especially to the mediastinum [2]. The prognosis is better if proper antibiotic and surgical debridement are promptly initiated.

## Case presentation

A 19-year-old male with no medical history is admitted for a complicated odontogenic infection secondary to a dental abscess. He refers to a one-week history of progressive fever, trismus, and facial pain. A head and neck tomography revealed an abscess in deep spaces of the neck. Ampicillin sulbactam was started, and a surgical plan was in place.

He underwent surgical drainage of the abscess with dental extraction; after 24 h, the culture taken at the operating room (OR) was positive for *Streptococcus anginosus*. Two days after the drainage, he still had a fever, and his condition worsened with shortness of breath and shock.

He was intubated and sent to the intensive care unit (ICU) for further care. The antibiotic treatment was adjusted to a broad spectrum with meropenem, vancomycin, and clindamycin under suspicion of a necrotic infection of the deep spaces of the neck.

**Figure 1:**
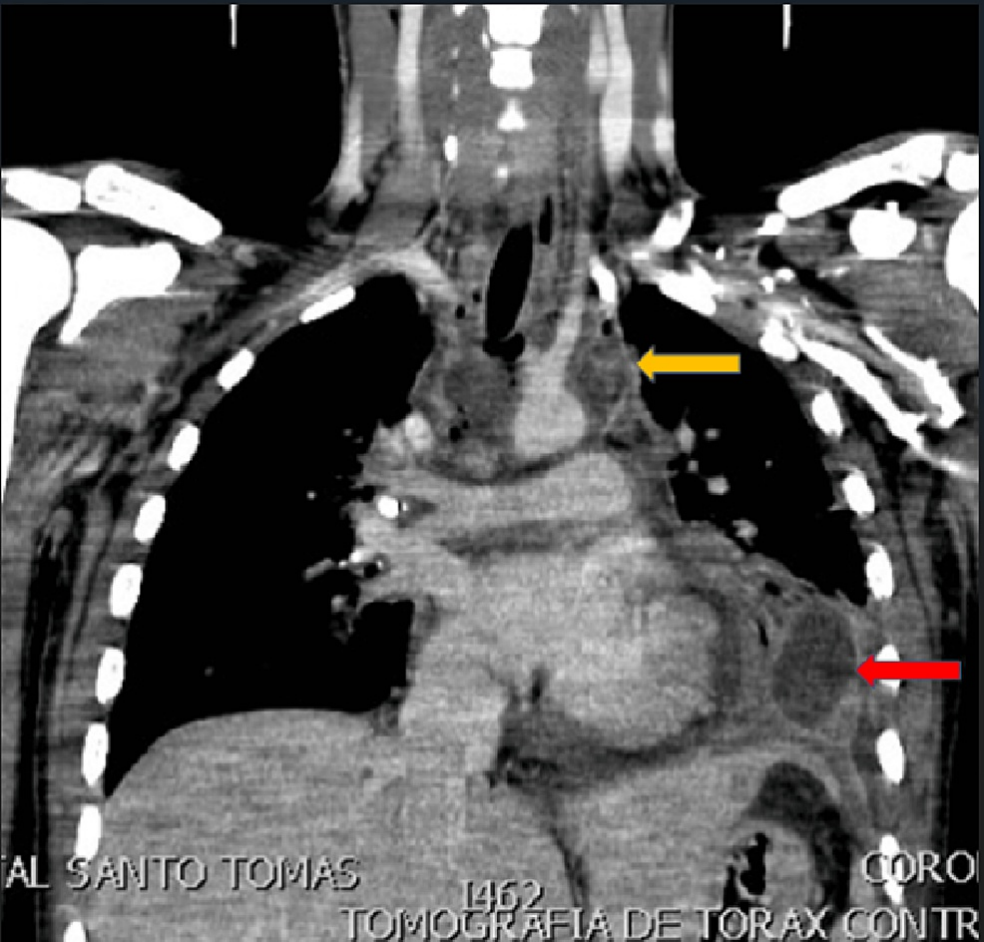
CT scan of the chest w/contrast, coronal view. Note mediastinal abscess (yellow arrow) and loculated empyema (red arrow).

**Figure 2:**
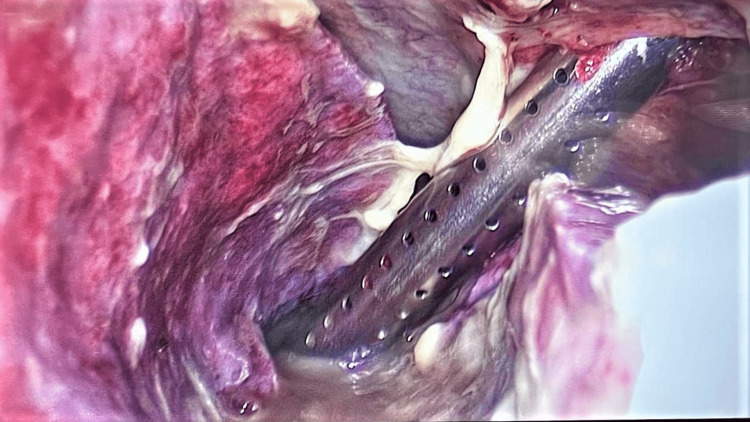
Video thoracoscopy (VATS). Photography shows empyema evacuation—samples were taken to the microbiology laboratory for cultures. VATS, video-assisted thoracoscopic surgery.

After admission to the ICU, the fever was persistent, and the weaning from the ventilator was unsuccessful. A follow-up CT scan of the neck and thorax was performed, where we found mediastinitis, mediastinal abscess, and an empyema (Figure 1). Because of this finding, he was again taken to the OR for video-assisted thoracic surgery, drainage of the mediastinal abscess, empyema, and early pulmonary decortication (Figure 2).

Tissue and fluid samples found are collected during the procedure and sent to a microbiology laboratory with an interest in anaerobes, cultured in blood agar, and then incubated in an anaerobic environment at 35-37°C. After incubation for five days, colonies grew. *Prevotella buccae* was identified with matrix-assisted laser desorption/ionization & time-of-flight (MALDI-TOF-MS) (Vitek MS).

After this procedure, he remained for approximately one month in the ICU, where he required an early tracheostomy and developed healthcare-associated infections (ventilator-associated pneumonia and candidemia) that were treated according.

Later, during his disease, pericardial effusion was found in a routine transthoracic echocardiogram. A subxiphoid pericardial window with a pericardial biopsy was done. We found a thickening of the pericardium. However, the cultures and histopathology were negative.

After five weeks in the ICU, he was successfully weaned from the ventilator, receiving meropenem and crystalline penicillin for 21 days then, he was transferred to the ENT ward, where he completed antibiotics and was sent home after 1.5 months of hospitalization.

## Discussion

Mediastinitis is a life-threatening condition that causes inflammation and infection of the connective tissue and structures within the mediastinum. Due to the critical contents of this area, infections can lead to high rates of morbidity and mortality, requiring comprehensive management in the intensive care unit by a multidisciplinary team. When managing mediastinitis it is crucial to define or classify the etiology which can be separated into three categories: deep sternal infection, esophageal perforation, and DNM. The infectious agents responsible for these entities are variable and change according to the infection’s acquisition mechanism which affects the medical and surgical approach (Table 1).

**Table 1:** Differences of the most common etiologies of mediastinitis Adapted from [3]. *S. aureus*, *Staphylococcus aureus*; *P. aeruginosa*, *Pseudomonas aeruginosa*; *S. pyogenes*, *Streptococcus pyogenes*; *H. influenzae*, *Haemophilus influenzae*; *S. pneumoniae*, *Streptococcus pneumoniae*; *M. catarrhalis*, *Moraxella catarrhalis.*

	Deep sternal wound infection	Eesophageal perforation	Descending necrotizing mediastinitis
Definition	Secondary infection of the mediastinum following a sternotomy.	Loss of integrity of the wall with spillage of content. It may be iatrogenic, spontaneous (Boerhaave’s syndrome), traumatic, and malignant.	Infection of the head and neck that spreads to the mediastinum via connective tissues.
Classification	Type 1 – skin and subcutaneous tissues. Type 2 – sternum or ribs. Type 3 – plus bone loss of sternum or ribs. Type 4 – complete affection of mediastinum.	-	Type 1 – Localized. Type 2 – Diffuse, extending into the lower mediastinum. Type 3 – Diffuse. Extending into both anterior and posterior lower mediastinum.
Microbiology	Monomicrobial (*S. aureus* > Enterobacteriaceas) anecdotal cases of non-tuberculosis mycobacteria. Other unusual causes: *Candida* spp. and other fungi. Nocardia.	Monomicrobial (normal oropharyngeal flora, *Streptococci*, *Neisseria*, *Prevotella*, *Fusobacterium*, among others) in critically ill patients exposed to broad-spectrum antibiotics: gram-negative aerobic bacteria, *Candida* spp. and *S. aureus*.	Polymicrobial variations according to the source: - Odontogenic: *Streptococcus* spp., *S. aureus*, *P. aeruginosa,* and *E. coli*. Relevant anaerobes are *Peptostreptococcus* spp., *Fusobacterium nucleatum*, *Prevotella* spp., and *Actinomyces* spp. -Pharynx: oral anaerobes, facultative streptococci (*S. pyogenes*); *H. influenzae.* -Otogenic: Streptococci, obligate anaerobes, *S. aureus*, and *P. aeruginosa*. - Chronic suppurative sinusitis: *S. pneumoniae*, *H. influenzae*, *M. catarrhalis*, *S. aureus*, and obligate anaerobes.

In the clinical case presented, the patient had a DNM, defined as an infection with its origin from a head and neck source, commonly from oropharyngeal or odontogenic focus, which spreads downward into the mediastinum [4]. The criteria established by Estera et al. for DNM are as follows: 1. Clinical manifestations of severe infection, 2. demonstration of characteristic tomographic features, 3. documentation of necrotizing mediastinal infection at operation, postmortem, or both; and 4. establishment of the relationship of oropharyngeal or cervical infection with the development of the mediastinal process.

One of the most important considerations for this diagnosis is the intraoperative findings by the surgeon (intraoperative characteristics of DNM): the presence of gray necrotic tissue, lack of bleeding, thrombosed vessels, foul-smelling pus, non-contracting muscle, and a positive “finger test" (lack of resistance to finger dissection in normally adherent tissues) [1].

DNM is caused by odontogenic infections (36-47%), pharyngeal (33-45%), or cervical infections (15%). In 6% of the cases, the cause is unknown [3]. These infections reach the mediastinum through the neck by three significant paths: 1. Pre-tracheal route to the anterior mediastinum, 2. lateral pharyngeal way to the medial mediastinum, and 3. retropharyngeal way to the posterior mediastinum [2]. 

DNM is an unusual infection and underestimated cause of death in the ICU. Identified risk factors associated with this disease are impaired immune function, diabetes, use of oral glucocorticoids and reduced tissue oxygenation caused by heart failure, intravenous drug abuse, alcoholism, respiratory insufficiency, and peripheral artery occlusive diseases [1-3].

Signs and symptoms of DNM are elusive and non-specific. Fever, chills, and tachycardia are commonly associated with the finding of an oropharyngeal/odontogenic infection. However, this can go unnoticed if the patient is intubated and sedated. Later, dyspnea, chest pain, and respiratory failure develop. If the origin is a head and neck infection, trismus can sometimes be found. Also, the laboratory's findings are not specific and can be summarized as elevated white cell count, C-reactive protein, and procalcitonin [3].

Because there is a lack of early clinical signs, once the physicians have a suspicion of DNM, contrast-enhanced computed tomography (CT) of the neck and chest is the imaging modality of choice to confirm the diagnosis [3]. CT findings include increased density of adipose tissues (>25 HU), cervical lymphadenopathy, mediastinal fluid collections, and pleural and pericardial fluid collections. In addition, myositis and vascular thrombosis development were there.

In general, the management of mediastinitis depends on the underlying etiology. It is a severe infectious disease that rapidly evolves into septic shock, an independent predictor of mortality [5]. Although empirical treatment is an urgent choice in managing DNM, microbiological sampling is mandatory through aspiration from deep abscess or debridement. Blood cultures should be collected too [3].

The choice of empirical antibiotics must cover aerobic and anaerobic bacteria associated with ENT and neck infections. Unfortunately, no clinical trials are evaluating the best treatment. Still, it is evident from a series of cases and expert opinions that the regimens described include broad-spectrum antibiotics: third-generation cephalosporin with metronidazole or a combination of piperacillin/tazobactam and clindamycin. Once the cultures are available, the antibiotic treatment must be tailored.

An exciting aspect of our clinical case is that the first culture of the cervical abscess debridement was positive for *Streptococcus anginosus*, which is the most common bacteria causing DNM and other deep neck spaces infection (*Streptococcus *spp) [6].

The isolation of *Prevotella buccae* from the mediastinal collection reflects the high value of automatized methods of microbiological diagnosis, allowing us to understand the nature of these infections and tailor the treatment. The identification of anaerobic bacteria has always been a challenge. However, mass spectrometry (MALDI-TOF) is now a reference resource for identifying these pathogens in clinical microbiology [7]. 

The duration of antibiotic treatment in DNM has yet to be established. The consensus is between 14 and 21 days. However, the course may be longer if complications appear. The other cornerstone in the management is surgical, which includes the treatment of the pharyngeal or dental focus and prompt and adequate drainage of the neck and mediastinum and surgical debridement, and airway management with an early tracheotomy [4].

Even if systematic debridement and broad opening of involved fascial spaces with proper antibiotic coverage are done, DNM is associated with elevated mortality (15-30%); the higher rates are associated with late diagnosis, inadequate surgical drainage of the mediastinum, advanced age, and elevated ICU severity scores [1,8].

## Conclusions

Head and neck infections can rapidly evolve with swift dissemination through the neck spaces until developing into life-threatening conditions like DNM.

Clinicians must be aware of the complexity of the physiopathology of this infection, and in patients with odontogenic/oropharyngeal infections, concerning symptoms must be considered for a rapid diagnosis.

DNM is associated with a significant burden of morbi-mortality, so the probability of success can increase with an early diagnosis, rapid initiation of an appropriate antibiotic (broad spectrum that covers bacteria associated with the original focus of infection), fast medical treatment and hemodynamic support after the onset of sepsis, and timely aggressive surgical treatment.
